# Glutamine energy substrate anaplerosis increases bone density in the Pah^enu2^ classical PKU mouse in the absence of phenylalanine restriction

**DOI:** 10.1002/jmd2.12308

**Published:** 2022-07-06

**Authors:** Steven F. Dobrowolski, Yu Leng Phua, Irina L. Tourkova, Cayla Sudano, Jerry Vockley, Quitterie C. Larrouture, Harry C. Blair

**Affiliations:** ^1^ Department of Pathology University of Pittsburgh, School of Medicine Pittsburgh Pennsylvania USA; ^2^ Division of Medical Genetics and Genomics Children's Hospital of Pittsburgh Pittsburgh Pennsylvania USA; ^3^ Pittsburgh Veteran's Affairs Medical Center Pittsburgh Pennsylvania USA; ^4^ Present address: Department of Genetics and Genomic Sciences Icahn School of Medicine at Mount Sinai New York USA

**Keywords:** glutamine, osteopenia, oxidative phosphorylation, Pah^enu2^, phenylketonuria

## Abstract

Osteopenia is an under‐investigated clinical presentation of phenylalanine hydroxylase (PAH)‐deficient phenylketonuria (PKU). While osteopenia is not fully penetrant in human PKU, the Pah^enu2^ mouse is universally osteopenic and ideal to study the phenotype. We determined Pah^enu2^ mesenchymal stem cells (MSCs) are developmentally impaired in the osteoblast lineage. Moreover, we determined energy dysregulation and oxidative stress contribute to the osteoblast developmental deficit. The MSC preferred substrate glutamine (Gln) was applied to enhance energy homeostasis. In vitro Pah^enu2^ MSCs, in the context of 1200 μM Phe, respond to Gln with increased in situ alkaline phosphatase activity indicating augmented osteoblast differentiation. Oximetry applied to Pah^enu2^ MSCs in osteoblast differentiation show Gln energy substrate increases oxygen consumption, specifically maximum respiration and respiratory reserve. For 60 days post‐weaning, Pah^enu2^ animals received either no intervention (standard lab chow), amino acid defined chow maintaining plasma Phe at ~200 μM, or standard lab chow where ad libitum water was a 2% Gln solution. Bone density was assessed by microcomputed tomography and bone growth assessed by dye labeling. Bone density and dye labeling in Phe‐restricted Pah^enu2^ was indistinguishable from untreated Pah^enu2^. Gln energy substrate provided to Pah^enu2^, in the context of uncontrolled hyperphenylalaninemia, present increased bone density and dye labeling. These data provide further evidence that Pah^enu2^ MSCs experience a secondary energy deficit that is responsive both in vitro and in vivo to Gln energy substrate and independent of hyperphenylalaninemia. Energy support may have effect to treat human PKU osteopenia and elements of PKU neurologic disease resistant to standard of care systemic Phe reduction. Glutamine energy substrate anaplerosis increased Pah^enu2^ bone density and improved in vitro MSC function in the context of hyperphenylalaninemia in the classical PKU range.

**Highlights**

- Pahenu2 osteopenia involves energy deficit.
- Glutamine energy substrate anaplerosis increases Pahenu2 mesenchymal stem cell functionality.
- An in vivo glutamine regimen increased Pahenu2 bone density independent of hyperphenylalaninemia.

## 1 INTRODUCTION

Phenylalanine hydroxylase (PAH)‐deficient PKU is a treatable inborn error of metabolism^1^, ^2^, ^3^, ^4^ where dietary Phe restriction is the principal intervention.^4^, ^5^, ^6^, ^7^ Early intervention, enabled by newborn screening, disallows neurologic devastation yet cognitive deficit, executive function deficit, neuropsychiatric phenotypes, and osteopenia remain common among treated patients.

Phenylketonuria (PKU) osteopenia was identified in the 1960s and clinical description of osteopenic PKU patients is extensive.^8^, ^9^, ^10^, ^11^ Lumbar spine bone mineral density Z scores of −2.0 are observed among early‐identified, continuously treated patients.^12^ Similar reduction in total body bone mineral density is observed.^13^ Equivalently low bone mineral density occurs in therapy noncompliant patients.^12^

Pathophysiological mechanisms of PKU bone disease remain ambiguous. The bone phenotype was originally attributed to diet therapy where by an undefined mechanism, bioavailability of calcium, phosphorous, and other bone‐forming material were reduced; however, this is poorly supported as osteopenia is recognized in patients that never received diet therapy and young patients following short‐term therapy.^12^ Several studies find no correlation^8^, ^11^, ^14^, ^15^, ^16^, ^17^ between hyperphenylalaninemia and bone disease; others show a negative correlation between hyperphenylalaninemia and bone disease.^28^, ^29^ Biochemical ambiguity does not end with Phe homeostasis as representation of bone formation markers,^18^, ^19^ bone resorption markers,^20^, ^21^ and other metrics related to bone provide no means to inform osteopenia risk.^18^, ^20^, ^22^

Phenylketonuria osteopenia has knowledge gaps as investigation is descriptive and pathophysiology is largely un‐investigated. While the bone phenotype is not fully penetrant in humans, osteopenia is universal in the Pah^enu2^ mouse model of classical PKU. Our previous investigations of Pah^enu2^ osteopenia identified a mesenchymal stem cell (MSC) developmental defect involving energy deficit and oxidative stress.^23^, ^24^, ^25^ These investigations were the first to assess bone development as a participatory element in the pathology of the PKU osteopenia. Here, we apply the MSC preferred energy substrate glutamine to in vitro MSC differentiation and mitochondrial oxygen consumption.^26^, ^27^, ^28^, ^29^ Gln enhances MSC osteoblast development and increases mitochondrial oxygen consumption. In vivo a post‐weaning Gln regimen increased Pah^enu2^ bone density. Moreover, augmentation of in vitro MSC metrics and in vivo bone density was achieved within the context of hyperphenylalaninemia in the classical range. Energy repletion provides an alternative intervention to treat Pah^enu2^ osteopenia acting independent of systemic Phe homeostasis.

## 2 METHODS

### 2.1 Pah^enu2^ and control animals

Pah^enu2^ and C57bl/6 were propagated at the Rangos Research Center animal facility at Children's Hospital of Pittsburgh with an approved protocol. Pah^enu2^ and control animals were generated matings heterozygous females and heterozygous males. Offspring were genotyped as described.^30^ Alternative homozygous genotypes (experimental enu2/enu2, control wt/wt) were used in experimental cohorts. After weaning (day of life 21), Pah^enu2^ animals were provided one of the following diets 1. Standard mouse chow; 2. standard mouse chow where the ad libitum water supply was a 2% Gln solution; 3. Phe‐free amino acid defined chow with Phe supplemented in drinking water (0.35 g/L). Standard chow and standard chow plus 2% Gln maintain Pah^enu2^ plasma of ~2000 μM in males and 2200 μM in females. Phe‐free amino acid defined chow produces Pah^enu2^ plasma Phe of ~200 μM.^30^ Animals provided dietary Phe restriction were utilized in in vivo histomorphometry studies. Owing to Gln aqueous instability, over the 60‐day post‐weaning regimen, a freshly dissolved 2% Gln solution was provided each Monday, Wednesday, and Friday. Control (wt/wt) littermates were provided standard mouse chow. Experiments used animals in the fed state. Animals (control, Pah^enu2^) were sacrificed by CO_2_ asphyxiation, at 2–3 months of age.

### 2.2 Mesenchymal stem cell culture and osteoblast differentiation

Experimental and control cohorts contained at least four animals (equal male/female representation). MSCs were prepared from 2‐ to 3‐month animals (control, Pah^enu2^) as described.^23^, ^24^, ^25^ Briefly, bone marrow was flushed (RPMI‐1640 10% fetal calf serum) with an insulin syringe from the femur and tibia. Aspirate was plated for ~18 h at 37°C to remove rapidly attaching fibroblast‐like cells. Non‐adherent cells were re‐plated at 2 × 10^6^ cells/cm^2^. After 72 h non‐adherent cells were discarded and adherent cells provided MSC proliferation medium (MesenCult Proliferation media, Stemcell Technologies). Cultures were expanded and passage 4–7 cells were used in osteoblast differentiation. Osteogenic differentiation applied for 14 or 21 days applied media containing 35 μg/ml L‐ascorbic acid, 10 mM β‐glycerophosphate, 10Pm adrenocorticotropic hormone, 10nM 1α, 25‐dihydroxyvitamin D3, and 0.5 mM CaCl_2._ ^31^ MSC osteoblast differentiation media in control Pah^enu2^ cultures is supplemented with Phe to 1200 μM. Pah^enu2^ MSC cultures receiving Gln energy substrate includes 1% Gln and Phe to 1200 μM. Over the course of differentiation, media were replaced every Monday, Wednesday, and Friday. MSC osteoblast differentiation was assessed by in situ alkaline phosphatase activity and mineralization. In situ alkaline phosphatase activity used 0.01% napthol AS‐MX substrate and fast blue product visualization as described.^23^, ^24^, ^25^ Visualizing mineralization used von Kossa silver staining as described.^23^, ^24^, ^25^ Densitometry analysis was performed after converting the image to grayscale, inverted, and the mean white area was measured with ImageJ software.

### 2.3 Oximetry of MSC cultures in osteoblast differentiation

Proliferating Pah^enu2^ and control MSCs (40 000 cells) were plated in 96‐well Seahorse oximetry plates. At confluence, osteoblast differentiation was induced as above. Experimental Pah^enu2^ cultures were provided media including 1% Gln. On day 14 post‐induction, 20 h prior to assessment, differentiating MSCs were washed and unbuffered media provided. Experimental conditions (supplemental Phe, Gln) were maintained in unbuffered media. Assessment utilized the Seahorse Mito Stress Test that applies oligomycin, carbonyl cyanide‐4‐phenylhydrazone (FCCP), 2‐deoxy‐glucose, and rotenone/antimycin to determine basal respiration, maximal respiration, ATP production, and spare capacity.^23^, ^32^, ^33^ During assessment, each experimental and control condition utilized eight replicate wells. Data analysis is as described using Graph pad software and unpaired T‐test.^23^, ^32^, ^33^

### 2.4 Microcomputed tomography and dye labeling

Experimental (Pah^enu2^ Phe restricted diet, Pah^enu2^ Gln energy substrate diet) and control (C57bl/6, unmanaged Pah^enu2^) animal cohorts (minimum 6 animals, equal male vs. female representation) were sacrificed 2 months post‐weaning. Trabecular bone analysis applied microcomputed tomography as described in a blinded manner.^24^, ^25^ Briefly, fixed lumbar vertebrae, were scanned in 70% ethanol at 6‐μm resolution with a 0.25‐mm aluminum filter; voltage and current were set at 69 kV and 100 μA. Image reconstruction utilized Nrecon and InstaRecon software. Cross sectional images used Dataviewer software. Quantitative analysis assessed a 1.2‐mm region at the midpoint of the fourth lumbar vertebrae. Determined were bone volume/total volume, bone surface density, trabecular number, and total porocity. Data represent that of the entire cohort (experimental, control) not parsed by sex. Dynamic histomorphometry applied 7 days and 2 days prior to sacrifice intra‐peritoneal injection of calcein (20 μg/g mouse weight) and xylenol orange (80 μg/g mouse weight), respectively. Cutting undecalcified 7 μm frozen sections applied tape means. Measurement of dye‐labeled surface was performed as described.^24^

## 3 RESULTS

### 3.1 In the context of hyperphenylalaninemia, Gln energy substrate increased Pah^enu2^ in situ alkaline phosphatase activity

**FIGURE 1:**
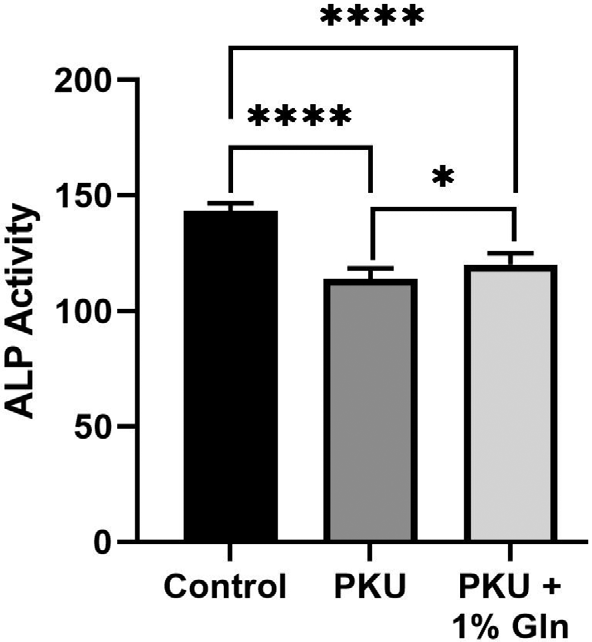
Gln increased in situ alkaline phosphatase activity in hyperphenylalaninemia FIGURE 1. MSCs from Pah^enu2^ (2 male and 2 female) and C57bl/6 (2 male and 2 female) were differentiated in standard media or media supplemented with 1% Gln. *p ≤ 0.05; ****p ≤ 0.0001

Figure 1 provides staining of in situ alkaline phosphatase activity. As previously reported, Pah^enu2^ MSCs in osteoblast differentiation have decreased alkaline phosphatase activity compared to C57bl/6. Pah^enu2^ MSCs provided Gln energy support demonstrate a statistically significant increase in in situ alkaline phosphatase activity. Mineralization did not increase with Gln energy substrate (data not shown).

### 3.2 In the context of hyperphenylalaninemia, Gln energy substrate increased Pah^enu2^ MSC oxygen consumption in osteoblast differentiation

**FIGURE 2:**
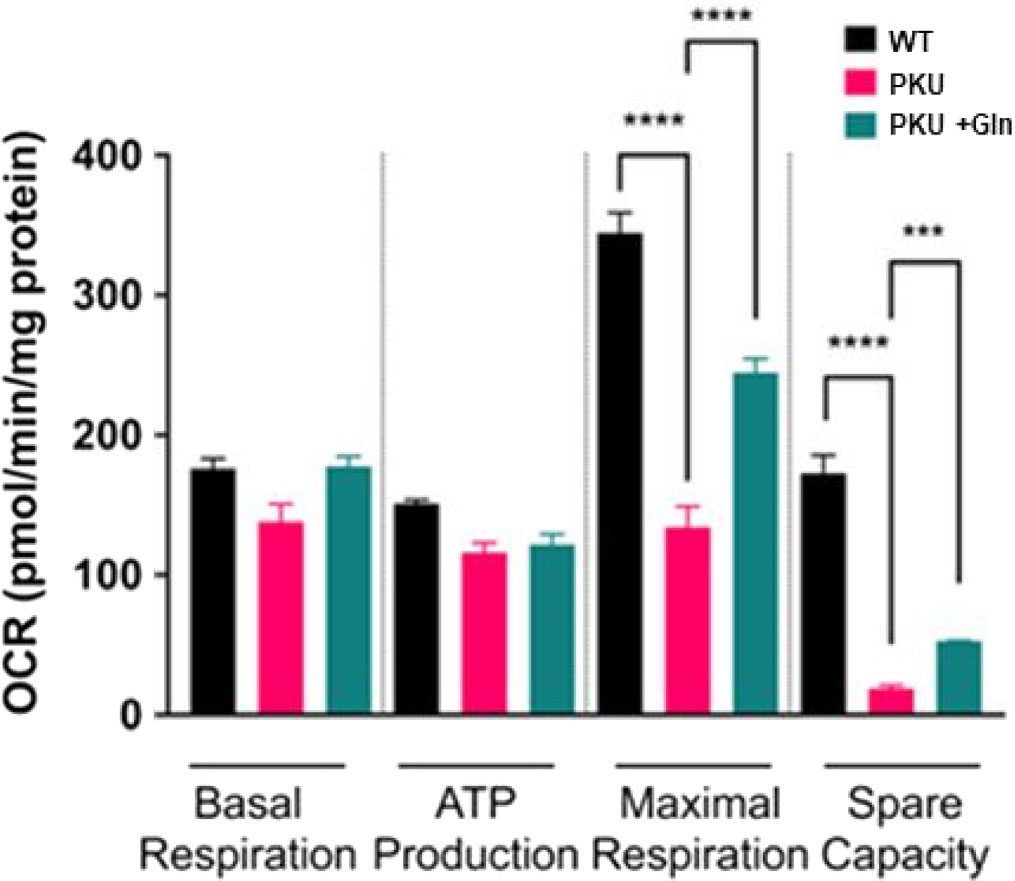
Gln substrate rescues mitochondria oxygen consumption in differentiating Pah^enu2^ MSCs in hyperphenylalaninemia FIGURE 2. Assessment of differentiating MSC from six Pah^enu2^ preparations (3 male and 3 female) and Six C57bl/6 (3 male and 3 female). Gln support of WT MSCs did not change respiration (data not shown); OCR, oxygen consumption rate. **** *p* ≤ 0.0001; *** *p* ≤ 0.001

Figure 2 oximetry applied the Seahorse Mito Stress Test to MSCs following 14 days of osteoblast differentiation. Basal oxygen consumption and ATP production are equivalent between C57bl/6, Pah^enu2^, and Pah^enu2^ provided Gln energy substrate. Maximum respiration and spare capacity of Pah^enu2^ and Pah^enu2^ with 1% Gln are both less than that of C57bl/6 controls. However, Gln energy substrate provided Pah^enu2^ MSCs statistically significant increases to both maximum respiration and spare capacity.

### 3.3 In vivo Gln energy substrate increases Pah^enu2^ bone density in uncontrolled hyperphenylalaninemia

In vivo assessment provided Pah^enu2^ Gln energy substrate from weaning (day of life 21) continuously for the following 60 days ad libitum as a 2% Gln solution in the water supply. These animals otherwise received normal chow causing unregulated Phe homeostasis of ~2000 in males and 2200 μM in females. For comparison, a separate Pah^enu2^ cohort was provided a Phe restricted diet, maintaining blood Phe of ~2200 μM. Microcomputed tomography, assessing the fourth and fifth lumbar vertebrae, determined bone density of untreated Pah^enu2^ and Phe‐restricted Pah^enu2^ is identical. All metrics (bone volume/total volume, bone surface density, trabecular number, and total porocity) are indistinguishable and significantly less than unaffected control littermates. Pah^enu2^ provided Gln energy substrate anaplerosis, in the context of unrestricted Phe homeostasis, show improved static histomorphometry metrics and improved dye labeling. Bone density improved among Phe unrestricted Pah^enu2^ receiving Gln energy substrate anaplerosis. Similarly, calcein and xylenol orange dye labeling was identical between control Pah^enu2^ and Phe‐restricted Pah^enu2^. Gln substrate increased dye labeling to equivalence with unaffected littermates.

## 4 DISCUSSION

Osteopenia is an under‐investigated phenotype in PAH‐deficient PKU and pathophysiology remains ill‐defined. PKU clinical phenotypes occur in brain and bone. As neither tissue express the PAH gene nor hydroxylate Phe, clinical phenotypes arise from secondary effect of biochemical dysregulation. The universally osteopenic Pah^enu2^ mouse is an ideal model to study osteopenia as the phenotype is not fully penetrant in patients. We determine Pah^enu2^ MSCs are deficient in osteoblast differentiation where energy deficit and oxidative stress are contributing factors.^23^, ^24^, ^25^ In the context of hyperphenylalaninemia, the MSC preferred energy substrate Gln increased in vitro alkaline phosphatase activity (a measure of osteoblast differentiation) and mitochondrial oxygen consumption (a measure of oxidative energy production). In vivo Gln energy substrate improved bone density concurrent to unregulated hyperphenylalaninemia.

**FIGURE 3:**
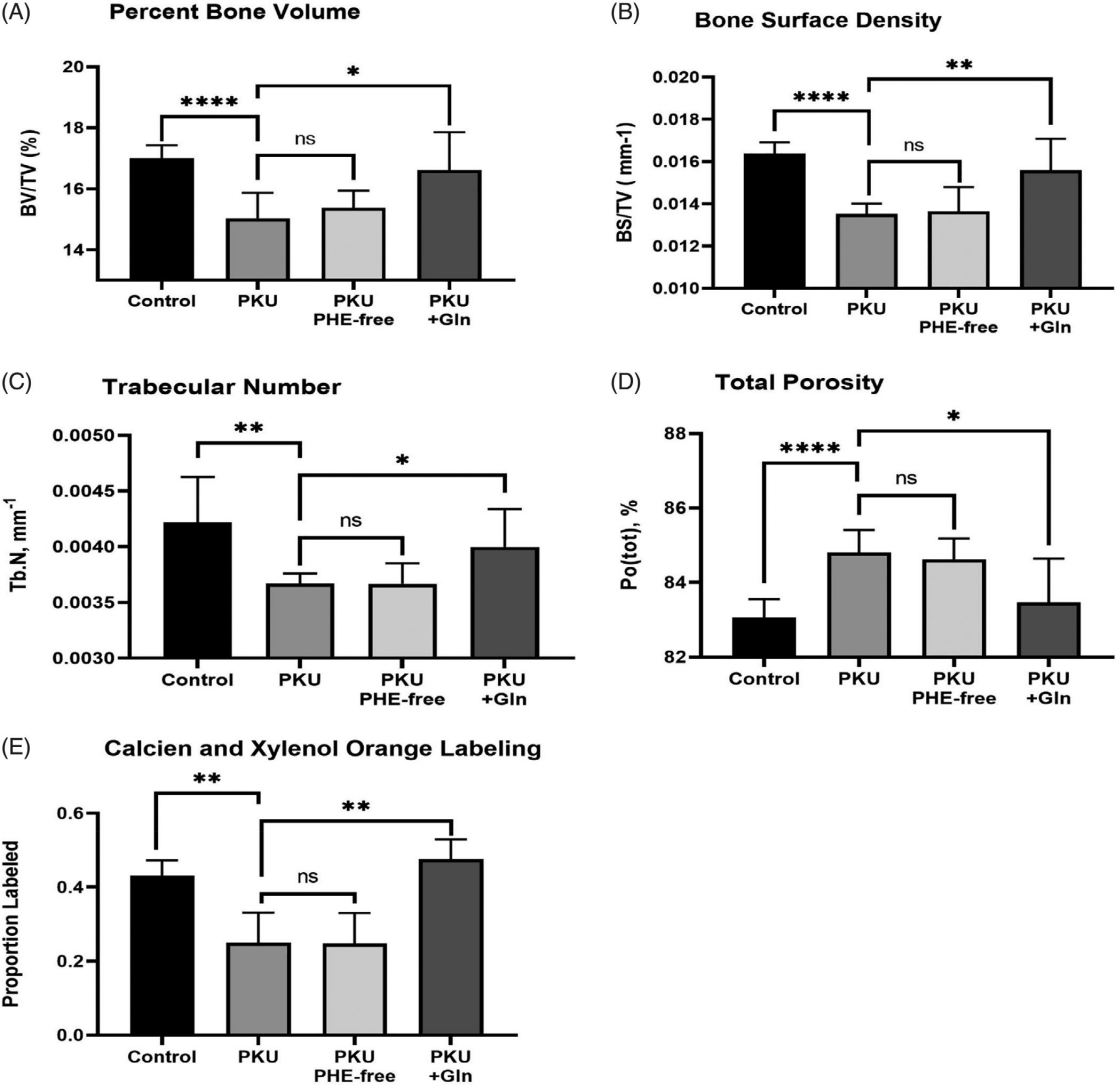
Increased in vivo Pah^enu2^ bone density and appositional growth with Gln energy substrate anaplerosis in uncontrolled hyperphenylalaninemia FIGURE 3. Static and dynamic histomorphometry of unmanaged Pah^enu2^ (plasma Phe 2000–2200 μM), Phe‐restricted Pah^enu2^ (plasma Phe 200uM) and Pah^enu2^ provided Gln energy substrate (plasma Phe 2000–2200 μM). Control animals are background strain C57bl/6 littermates. All cohorts have a minimum of six animals (3 male and 3 female). Static histomorphometry metrics determined in third or fourth lumbar vertebrae: (A) Percent bone volume, (B) Bone surface density, (C) Trabecular number, (D) Total porocity. Dynamic histomorphometry appositional dye labeling determined in third lumbar vertebrae: 3E In vivo calcein and xylenol orange labeling. * *p* ≤ 0.05; ** *p* ≤ 0.01; **** *p* ≤ 0.0001

While resting and proliferating MSCs are glycolytic, those in the course of osteoblast differentiation require oxidative phosphorylation. Respirometry applied to mitochondria from Pah^enu2^ MSC during osteoblast differentiation, showed an attenuated complex 1 response to pyruvate substrate; however, complex 1 response to glutamate (Glu) substrate was similar to controls.^23^ Respirometry in Pah^enu2^ brain tissue mitochondria showed similar attenuated response to pyruvate substrate.^34^ Pyruvate enters mitochondria through voltage dependent anion channels. The Phe catabolite phenylpyruvate is structurally similar to pyruvate and a recognized inhibitor of pyruvate mitochondrial transport.^35^, ^36^, ^37^, ^38^ Pyruvate transport inhibition reduces acetyl‐CoA that drives Kreb cycle processivity and creation of reducing equivalents for oxidative phosphorylation. On the plasma membrane, MSCs express SLC1A5 high‐affinity Gln transporter. Cytosolic glutaminase converts Gln to Glu. Two related proteins, the mitochondrial glutamate carriers GC1 (SLC25A22) and GC2 (SLC25A18) transport Glu into the mitochondria, where it is converted to alpha‐ketoglutarate distally repleting the Kreb cycle. We posit Gln energy substrate increases Pah^enu2^ bone density by alternative pathway Kreb cycle repletion circumventing phenylpyruvate inhibition of pyruvate transport. Gln up‐regulates energy homeostasis enabling osteogenesis (Figures 1, 2, 3). Augmented MSC function and increased bone density within the context hyperphenylalaninemia is a consequence of alternative energy pathway utilization. Moreover, realizing increased bone density in uncontrolled hyperphenylalaninemia strongly argues against causation by asymmetric amino acid transport through the LAT1 (SLC7A5 gene product) transporter.

Increased bone density is the first physiologically quantifiable PKU intervention response occurring independent of systemic Phe management. As most adolescent/adult PKU patients are therapy noncompliant, Gln energy support may provide a means to treat osteopenic patients unwilling/unable to engage systemic Phe reduction. Further, even more importantly, these data suggest energy deficit may contribute to PKU neurologic phenotypes. Even that small minority of adult/adolescent PKU patients that remain compliant to Phe reduction therapy present cognitive decline, executive function deficit, and other late onset neurologic phenotypes. Should energy deficit contribute to neurologic disease, energy support, be it Gln or other alternative pathway substrates, may provide an under‐appreciated intervention opportunity.^39^

Literature Cited.

## FUNDING INFORMATION

Veteran's Affairs 2I01 BX002490‐06A1 and 1R01AR076146‐01A1 to HCB.

National PKU Alliance to SFD.

## CONFLICT OF INTEREST

No authors have competing interests related to these studies.

## ETHICS STATEMENT

No patients were involved in these studies.

## INSTITUTIONAL COMMITTEE FOR CARE AND USE OF LABORATORY ANIMALS

Pah^enu2^ and C57bl/6 animals are managed under an approved protocol of the Children's Hospital of Pittsburgh IACUC.

## Data Availability

Data will be made available upon reasonable request.
